# Alanine Mutagenesis Identifies Specific Amino Acids of Nemertide Alpha‑1 Activity and Its Binding to Target Receptors

**DOI:** 10.1021/acs.jnatprod.5c01177

**Published:** 2025-12-17

**Authors:** Quentin Laborde, Steve Peigneur, Erik Jacobsson, Ulf Göransson, Jan Tytgat, Håkan S. Andersson

**Affiliations:** † Pharmacognosy, Department of Pharmaceutical Biosciences, Biomedical Center, 8097 Uppsala University, Box 574, SE-751 23, Uppsala, Sweden; ‡ Toxicology & Pharmacology, University of Leuven (KU Leuven), O&N 2, PO Box 922, Herestraat 49, 3000, Leuven, Belgium; § University of Vienna, Faculty of Chemistry, Institute of Biological Chemistry, Währinger Straße 38, 1090, Vienna, Austria; ∥ Department of Medical Biochemistry and Biophysics, 27106 Karolinska Institutet, 17177 Stockholm, Sweden

## Abstract

We recently discovered and characterized a novel family of peptide toxins, the alpha-nemertides, from the marine ribbon worm *Lineus longissimus*. These 31-residue peptides show potent neurotoxicity against invertebrate voltage-gated sodium (Na_v_) channels, making them promising candidates for biopesticide development. To explore structure–activity relationships, we synthesized 20 nemertide alpha-1 mutants (17 alanine, 3 lysine substitutions) to identify residues critical for activity and selectivity. Key positions, including S12, T17, N19, W22, and F24, were found to influence activity on Na_v_ channels significantly. Notably, the S12A mutant showed high selectivity for invertebrate Na_v_s, suggesting its potential as a selective tool or lead scaffold. Our findings highlight critical interaction points likely to be involved in binding to site 3 of domain IV on Na_v_ channels and demonstrate how targeted modifications can sharpen selectivity. These insights support the rational design of more selective peptides and identify S12A as a promising candidate for further development as a biopesticide.

## Graphical abstract

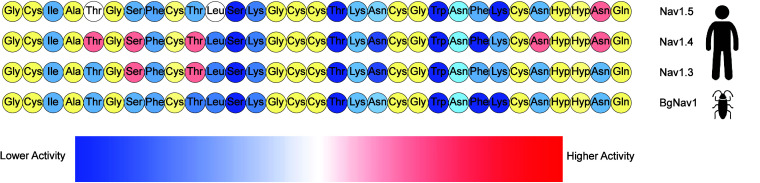

Alanine scanning, also named methyl scanning, is a well-established technique to study peptide structure–activity relationships (SAR).^1,2^ It involves systematic replacements of amino acids of interest by alanine, replacing side chain groups with a methyl group.^1^ Alanine is chosen due to its chemical stability, low steric hindrance, and absence of charge. The conformational impact from this systematic replacement is expected to be neutral or very low.^1^ Additionally, the substitution of side chain groups by methyl groups is a relevant procedure to identify activity (or loss thereof) and binding sites.^2^ Hence, this method has proven suitable to investigate essential residues for activity and binding to ion channels.^3,4^ Alanine scanning has previously been used successfully to elucidate selectivity and/or affinity of several peptide toxins with protein receptors from scorpion, spider, and sea anemone peptide toxins.^5–12^

Recently, we described and characterized the presence of a new family of peptide toxins named alpha-nemertides in the epidermal mucus of *L. longissimus*.^13,14^ These toxins exhibited potent effects on invertebrate Na_v_ channels with EC_50_ in the low nanomolar range (*Blattella germanica*: 8.6 nM). The ecological roles of these peptides are yet to be elucidated, and the investigation of their potential use is still ongoing. Previous studies^13–16^ have suggested possible application of nemertide alpha-1 as a biopesticide and in channelopathy treatment, respectively. Nemertide alpha-1 could potentially also be utilized as a tool allowing a better understanding of voltage-gated ion channels.

Transcriptomic analysis of nemerteans of the Lineidae lineage resulted in the discovery of seven alpha-nemertide sequences.^13^ These alpha-nemertides were synthesized and their activity was assessed *in vivo* using *Artemia salina* and *in vitro* using whole cell patch assay of a panel of Na_v_ channel subclasses.^14^ These analyses demonstrated the importance of Lys4 and Phe8 for bioavailability and effect on the channel, respectively.^14^ In light of these findings, we selected alanine scanning to gain a better understanding of nemertide alpha-1 and to identify the amino acid positions that are decisive for defining the affinity and selectivity of nemertide alpha-1 for VGSC.

In this project, we report the synthesis, oxidative folding, and toxicity characterization of 20 nemertide alpha-1 mutants (Figure 1). Toxicity data on *Artemia salina* and Na_v_ channels from vertebrates and invertebrates underline each amino acid’s activity and selectivity contribution in the structure–activity relationships of the peptide. A mutant displaying a pronounced enhancement of selectivity for BgNav (S12A) was then subjected to analysis over a wider panel of VGSCs.

**Figure 1:**
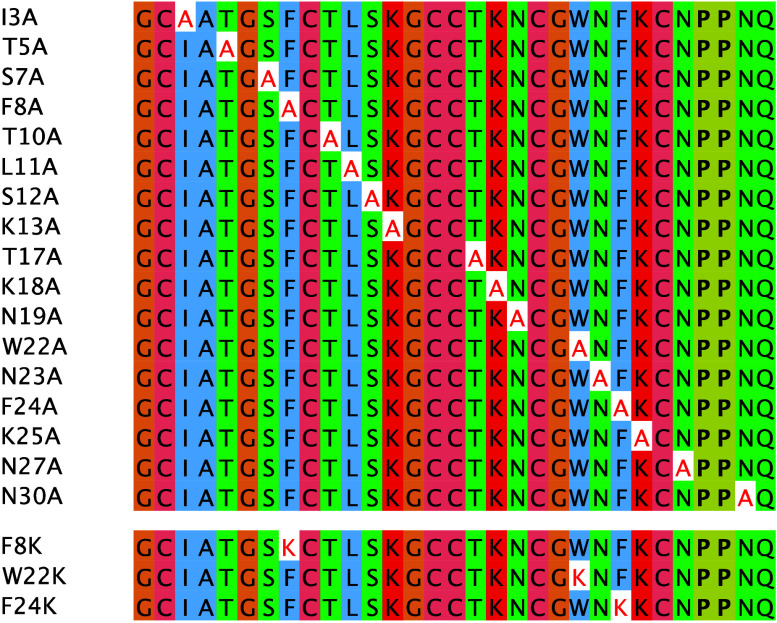
Sequence alignment of alanine mutant sequences and lysine mutant sequences. Alanine (A) and lysine (K) mutations are annotated with red letters. Hydroxyproline is annotated with bold letters (P). Alignment was performed with Muscle^17^ and coloring with ClustalW^18^ in Jalview,^19^ used with permission.

## Results and Discussion

A total of 17 alanine and 3 lysine mutant peptides were successfully synthesized. However, certain mutants—specifically, L11A, S12A, K13A, and T17A—proved challenging and required multiple synthesis attempts before yielding acceptable products. To overcome synthesis difficulties, a strategic substitution was made: the Fmoc-Gly-Cys­(ψ­(Dmp,H)­pro)-OH dipeptide was used at positions 14 and 15 instead of the standard Fmoc-Leu-Ser­(ψMe,MePro)-OH at positions 11 and 12. The use of pseudoproline dipeptide, which was previously introduced in α-1 synthesis to mitigate chain aggregation, facilitated successful peptide assembly.^13^ Crude yields following synthesis ranged between 120 and 140 mg (approximately 70–80%), which was lower than theoretical expectations. Despite achieving high relative purity (>95%), final yields after oxidative folding and purification were notably low, ranging from 0.42 to 4.70 mg—equivalent to 0.24–2.75% of the theoretical yield (Table 1, Figure S1). Experimental molecular masses for each mutant were confirmed via deconvoluted (3z) mass spectra (Table S1).

**Table 1:** Theoretical Yields, Final Yields Postfolding and Purification, and the Percentage Yield Relative to Theoretical Expectations^a^

Peptide	Theoretical Yield (mg)	Final Yield (mg)	Percentage Yield (%)
I3A	172	0.57	0.33
T5A	173	0.45	0.36
S7A	173	0.42	0.24
F8A	170	3.90	2.29
T10A	173	0.28	0.16
L11A	172	1.34	0.78
S12A	173	1.81	1.04
K13A	171	0.86	0.50
T17A	173	3.76	2.18
K18A	171	4.70	2.75
N19A	172	0.36	0.21
W22A	168	0.65	0.38
N23A	172	0.50	0.29
F24A	170	3.16	1.86
K25A	171	1.32	0.77
N27A	172	0.71	0.42
N30A	172	2.42	1.41
F8K	163	2.54	1.56
W22K	165	3.40	2.06
F24K	163	1.47	0.90

a Final yields were quantified using a Direct Detect infrared spectrometer.

The synthesis, folding, and evaluation of 20 peptide mutants is a substantial undertaking, and certain methodological compromises were made to streamline the process. A uniform SPPS protocol was applied to all mutants, foregoing individual optimization to save time. This likely contributed to the reduced yields observed for some sequences, as SPPS efficiency is highly sequence-dependent. Amino acid substitutions can significantly affect synthesis outcomes due to steric hindrance and variable coupling efficiencies. To address these challenges, pseudoproline dipeptides were incorporated to enhance synthesis success, particularly for α-1 mutants.

Following extensive purification and oxidative folding, the final yields remained low (Table 1). This outcome can be attributed to peptide loss during purification and the limited efficiency of oxidative folding. For example, a 30 mg peptide batch typically yielded no more than 3 mg of correctly folded product. As with synthesis, individual optimization of folding conditions was deemed impractical due to time constraints.

To improve folding efficiency, an alternative method was introduced starting with alpha-1. Misfolded peptides were reduced using TCEP for 1–2 h to cleave disulfide bonds.^20–22^ The reduced peptides were then separated from TCEP by RP-HPLC UV and lyophilized. These lyophilized peptides were subsequently subjected to a second round of oxidative folding, allowing to improve the yields of correctly folded products. The low yields represent a challenge in terms of upscaling, although this was not the focus in this study. We perceive that upscaling may be more successfully realized using biological expression systems than SPPS.

The hypothesized mechanism of action of alpha-nemertides relies on previous electrophysiology measurements of the action of alpha-1 on Na_v_s, which suggested that toxin binding promoted inhibition of inactivation of the ion channels.^13^ This was found indicative of site 3 binding,^23^ suggesting a mode of action akin to alpha-scorpion toxins and certain sea-anemone venoms albeit with very low structural resemblance to alpha-nemertides.^13^

EC_50_ values were evaluated for the 20 mutants *ex vivo* on selected Na_v_ channels and *in vivo* on *Artemia salina* (Table 2). Activity profiles are shown in Figure 2. Almost all mutants exhibited a higher EC_50_ than that of alpha-1 EC_50_ (1.2 μM), except for I3A, T5A, and N30A (1.2 μM, 0.53 μM, and 0.25 μM, respectively). Five Ala mutants (S12A, T17A, K18A, W22A, and K25A) and all three Lys mutants were inactive at the maximum tested concentration (60 μM).

**Figure 2:**
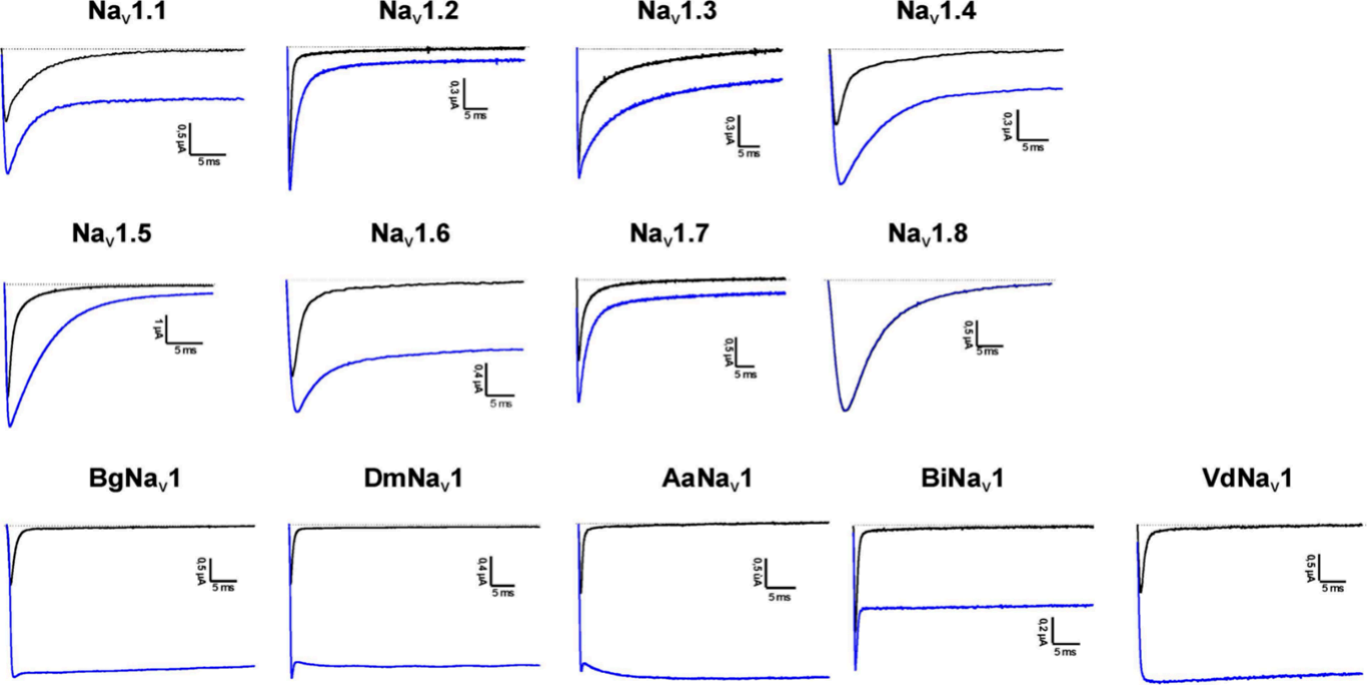
Activity profile of α-1 on vertebrate and invertebrate Nav channels. Dotted line indicates zero-current level; black: current traces in control conditions; blue: steady-state current traces after application of 1 μM toxin.

**Table 2:** EC_50_ Values of Alpha-Nemertide Mutants in Na_V_1.3–1.5 and BgNav1 (*ex vivo*, nM) and *Artemia* *salina* (*in vivo*, μM)^a^

Peptide	BgNav1	Nav1.3	Nav1.4	Nav1.5	Artemia
Alpha-1	8.6 ± 2.9	135.4 ± 76.3	145.5 ± 47.5	138.3 ± 25.5	1.2 ± 0.15
I3A	49.7 ± 9.1	186.3 ± 32.4	375.8 ± 82.8	701.4 ± 115.9	1.2 ± 0.47
T5A	45.6 ± 3.7	127.2 ± 43.7	112.9 ± 16.9	142.5 ± 43.3	0.53 ± 0.98
S7A	35.9 ± 13.3	93.4 ± 14.7	90.6 ± 37.0	146.6 ± 31.5	2.2 ± 3.2
F8A	68.7 ± 12.8	458.6 ± 61.8	818.1 ± 31.7	250.3 ± 32.3	7.7 ± 1.1
T10A	155.4 ± 131.7	418.9 ± 92.4	117.1 ± 51.1	110.2 ± 9.4	5.2 ± 1.5
L11A	298.2 ± 35.2	201.5 ± 47.9	704.2 ± 57.4	140.5 ± 18.0	19.0 ± 0.72
S12A	281 ± 75.0	n.a	n.a	n.a	n.a
K13A	386.3 ± 32.4	379.4 ± 91.0	5514.1 ± 154.5	595.1 ± 20.4	25.0 ± 1.5
T17A	825.9 ± 4.0	n.a	n.a	n.a	n.a
K18A	32.9 ± 10.9	675.4 ± 15.1	667.1 ± 17.9	373.1 ± 152.7	n.a
N19A	19.2 ± 7.3	n.a	n.a	534.2 ± 60.9	9.2 ± 18
W22A	2004 ± 188.0	n.a	n.a	n.a	n.a
N23A	19.5 ± 3.4	183.9 ± 37.6	229.6 ± 14.6	307.1 ± 12.5	1.5 ± 2.9
F24A	1046.5 ± 196.1	779.2 ± 77.3	n.a	577.1 ± 28.8	21.0 ± 3.4
K25A	454.7 ± 69.4	445.7 ± 52.4	919.8 ± 79.7	n.a	n.a
N27A	48.3 ± 2.8	465.7 ± 21.3	58.3 ± 28.2	320.0 ± 163.8	5.6 ± 4.8
N30A	137.9 ± 6.2	2759.5 ± 458.4	99.0 ± 26.7	167.3 ± 66.5	0.52 ± 0.25
F8K	91.9 ± 8.4	467.2 ± 64.9	1203.5 ± 147.2	223.9 ± 6.33	n.a
W22K	1051.0 ± 178.7	n.a	n.a	n.a	n.a
F24K	599.6 ± 86.7	n.a	n.a	n.a	n.a

a n.a: not active at the highest tested concentration (50 μM for electrophysiology measurements and 60 μM for *Artemia salina* measurements).

The results were summarized in a heatmap, and mutant EC_50_ values were normalized per column to the EC_50_ value of alpha-1 for each channel (BgNa_v_1: 10.3 nM, Na_v_1.3: 135.4 nM, Na_v_1.4: 184.0 nM, Na_v_1.5: 138.3 nM, and Artemia: 1.2 μM); this is expressed as a normalized value of 1.0 on the first row of the figure for each channel (Figure 3).

**Figure 3:**
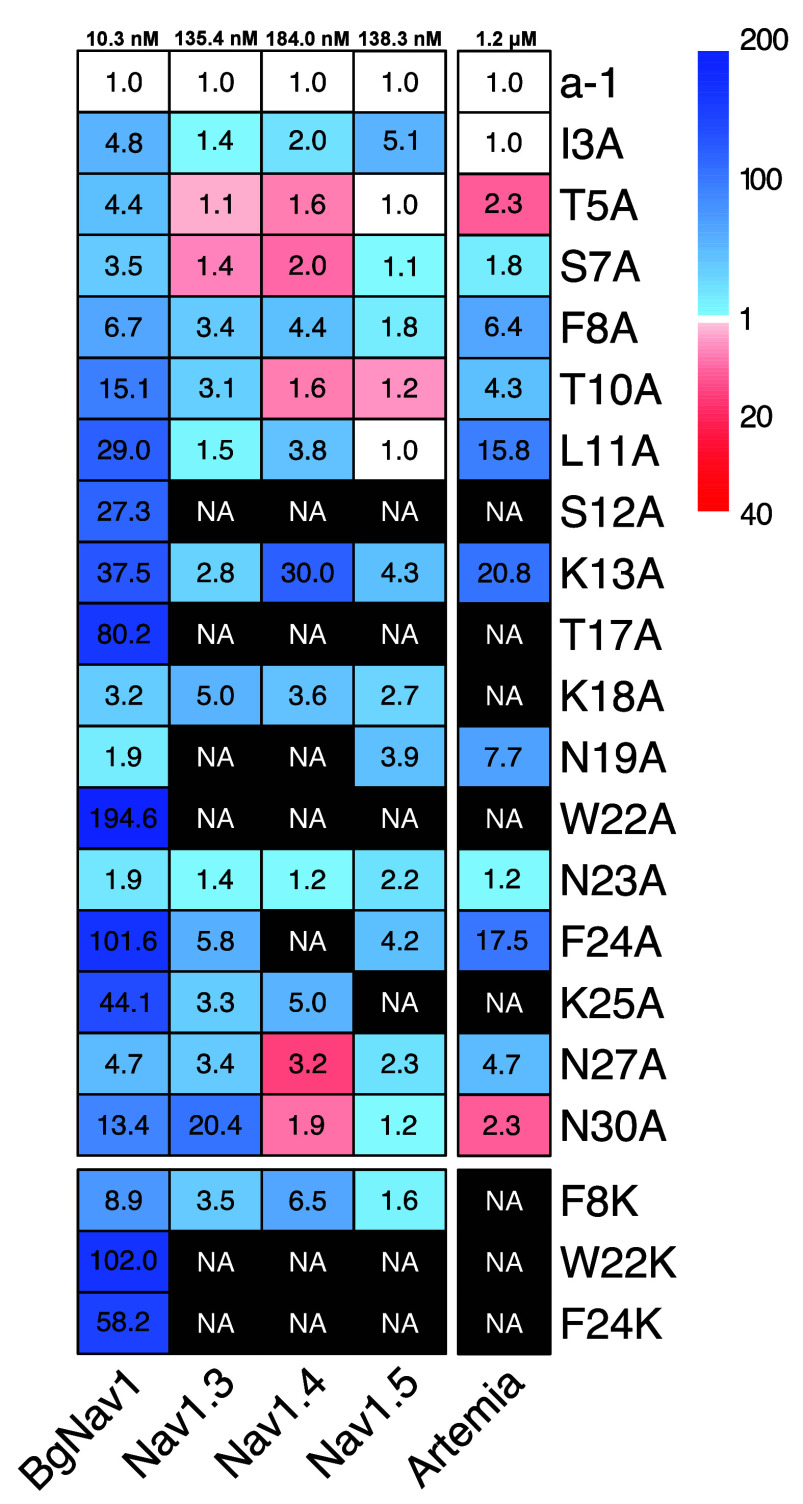
Heatmap of the normalized EC_50_ from alpha-1, the alanine and lysine mutants on BgNa_v_1, Na_v_1.3, Na_v_1.4, and Na_v_1.5, and *Artemia salina*. White cells represent mutants exhibiting activity comparable to that of alpha-1. Blue cells (light blue to dark blue) represent mutants with lower activity than alpha-1. Red cells (light red to dark red) represent mutants with higher activity than alpha-1. Black cells represent mutants with no activity compared to a-1. Values indicate how many folds the mutant differs from alpha-1. Each column has been normalized to a-1 EC_50_ for each channel. Scales are different between the blue (1 to 200) and the red part (1 to 40). NA: Not active at the highest tested concentration (50 μM for electrophysiology measurements and 60 μM for *Artemia salina* measurements).

An overall observation of the electrophysiology measurements is that Ala substitutions at S12, T17, and W22 resulted in deleterious effects across all investigated Na_v_ channels. K13A also exhibited complete loss of, or markedly lower, activity on all Na_v_ channels, albeit less pronounced (Figure 3). In contrast, the N-terminal region—specifically positions I3, T5, S7, F8, F10, and to some extent L11—showed relative insensitivity to Ala substitution. This insensitivity was similarly observed at positions 23 and 27 in most cases.

All peptides demonstrated activity on BgNa_v_1; however, their activities were consistently lower than that of nemertide alpha-1 (EC_50_ 8.6 ± 2.9 nM). The most significant effects of Ala mutations were observed for T17A, W22A, F24A, and K25A, with a 44- to 195-fold increase in EC_50_. In contrast, the mutations I3A, T5A, S7A, N19A, N23A, and N27A exhibited relatively small effects, with a 1.9- to 6.7-fold increase in EC_50_.

In the case of Na_v_1.3, two mutations (T5A and S7A) appeared to enhance activity compared to alpha-1, with a 1.1- and 1.4-fold reduction of EC_50_, respectively, in relation to 135.4 ± 76.3 nM for alpha-1. Conversely, activity was reduced for several mutants, including K18A, F24A, and N30A (5- to 20-fold increase in EC_50_), and S12A, T17A, N19A, and W22A, exhibiting no detectable activity.

For Na_v_1.4, five mutants (T5A, S7A, T10A, N27A, and N30A) exhibited lower apparent EC_50_ values than that of alpha-1 (184 ± 65.4 nM), ranging from 1.6- to 3.2-fold reduction. A pronounced negative impact on affinity was observed for F8A, L11A, K13A, K18A, and K25A (3.8- to 30-fold increase in EC_50_). Ala mutants S12A, T17A, N19A, W22A, and F24A exhibited no detectable activity.

In the case of Na_v_1.5, four mutants exhibited EC_50_ values in the same range as alpha-1 (T5A, S7A, L11A, and N30A), whereas 1.2-fold higher activity was evident for T10A. I3A, K13A, N19A, and F24A were clearly unfavorable mutations for Na_v_1.5 activity, and no detectable activity was observed for S12A, T17A, W22A, or K25A.

A subset of the mutants was also analyzed for their activity on Na_v_1.1, Na_v_1.2, and Na_v_1.6 (Figure 4A). This included the N-terminal mutants (T5, S7, F8, F10), three midsequence mutants (N19A, W22A, N23A), and three C-terminal mutants (F24A, N27A, N30A). In the case of Na_v_1.1, especially F8A (EC_50_ 58.9 ± 23.5 nM) stood out as more active than alpha-1 (EC_50_ 124.1.9 ± 28.7 nM), and T5A, S7A, T10A, and N27A as well as N30A displayed activity in the same range as alpha-1, whereas very low activity was evident for W22A and no activity at all for N19A and F24A. In the case of Na_v_1.2, T10A was the most active peptide (EC_50_ 267.6 ± 41.2 nM versus 359.6 ± 89.8 for alpha-1), whereas T5A and N27A displayed activity in the same range as alpha-1. No activity was observed for N19A, W22A, F24A, or N30A. In addition to the above, I3A, S7A, T10A, N27A, and N30A were analyzed for their activity on Na_v_1.7 (Table S2). All mutants displayed in the range 1.5–3 times lower activity than alpha-1 (EC_50_ 75.6 ± 33.9 nM), except for N27A and N30A, which displayed lower activity by an order of magnitude.

**Figure 4:**
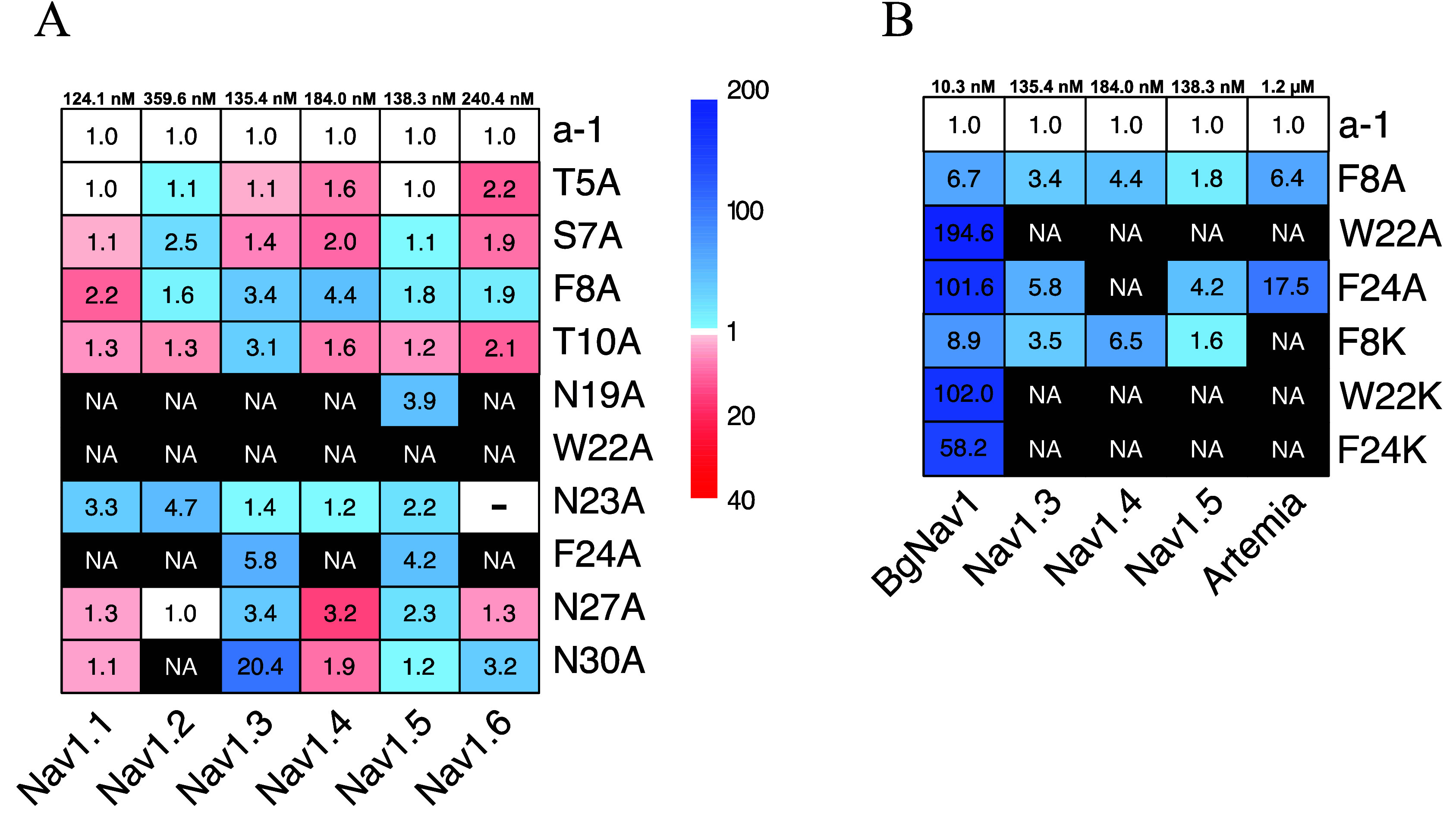
(A) Heatmap of the normalized EC_50_ from alanine mutants on Na_v_1.1 to Na_v_1.6. (B) Heatmap of the normalized EC_50_ from alanine and lysine mutants on BgNa_v_1 and Na_v_1.3 to Na_v_1.5. White cells represent mutants sharing the same activity as alpha-1. Blue cells (light blue to dark blue) represent mutants with a lower activity than alpha-1. Red cells (light red to dark red) represent mutants with a higher activity than alpha-1. Black cells represent mutants with no activity compared to alpha-1. Values indicate how many folds the mutant differs from α-1. Each column has been normalized to alpha-1 EC_50_ for each channel. Scales are different between the blue part (1 to 200) and the red part (1 to 40). NA: Not active at the highest tested concentration (50 μM for electrophysiology measurements and 60 μM for *Artemia salina* measurements).

The three additional Lys mutants (F8K, W22K, and F24K) were selected on account of the aromatic side chains of the native peptide (Figure 4B). W22K and F24K displayed a 102- and 58-fold increase in EC_50_, respectively, for BgNa_v_1, and no activity for any other Na_v_s. The effect of F8K substitution proved less detrimental for activity, with only an 8.9-fold reduction of affinity on BgNa_v_1, and less so for mammal Na_v_s (1.6- to 6.5-fold reduction). Whereas none of the Lys mutants were active on *Artemia*, it is noteworthy that they were all more active on BgNa_v_1 than the corresponding Ala mutants.

The main purpose of alanine scanning was to pinpoint key positions for shaping the selectivity. As previous work^13^ has indicated that natural targets of alpha-1 reside within *Arthropoda*, activity on BgNa_v_1, as well as *in vivo* experiments on *Artemia*, was deemed of special relevance. However, some mutations appear to shift selectivity favorably toward certain mammalian Na_v_s, forming starting points for the development of Na_v_ subclass-specific toxins.

A general observation is that both *Artemia* and electrophysiology results indicate a few key positions for shaping selectivity: S12, T17, N19, W22, and F24 (Figure 5). For the individual Na_v_ channels the following observations are warranted in this context: (Table S3, Figure S3). **BgNa**_**v**_ **1:** S12A has 33 times lower activity than native alpha-1, but it displays no activity for any of the other systems studied. The fact that EC_50_ is still low for this mutant (281 ± 75 nM) points to S12 as a target for further adaptation of the alpha-1 framework for insecticidal purposes. The same is true also for T17A, although a rather low activity is observed on BgNa_v_1 (825.9 ± 4 nM). Similarly, N19A is inactive in all but two systems; activity is only mildly negatively impacted on BgNa_v_1 (19.2 ± 7.3 nM), but high activity is retained on Na_v_1.5. **Na**_**v**_ **1.1:** Although the panel of mutants analyzed was smaller for this channel, some results stand out: F8A is the most active of the 11, whereas activity was negatively impacted in all other systems. Also, N27A and N30A showed slightly higher affinities for Nav1.1 than the native alpha-1, but activities on Na_v_1.4 (both) and Na_v_1.5 (N30A) were also favored. A very low EC_50_ was also observed for T10A, but this mutation had similarly high activities on several other Na_v_ subclasses. **Na**_**v**_ **1.2:** Both T10A and N27A showed improved affinities for Na_v_1.2, but significant cross-selectivity was evident for Na_v_1.5 and Na_v_1.4, respectively. **Na**_**v**_ **1.3:** Both I3A and K13A favored selectivity, and T10, L11, and N23 appeared similarly favorable, although certain cross-selectivity was observed (T10, L11: Na_v_1.5; N23: *Artemia*). **Na**_**v**_ **1.4:** No mutations appeared to be exclusively favorable for Na_v_1.4 selectivity. Favorable positions were S7, N27, and N30, although cross-selectivity was evident in all cases (Na_v_1.3, Na_v_1.1, and Na_v_1.1+Na_v_1.5, respectively). **Na**_**v**_ **1.5:** Two mutants (T5A and N27A) showed similar and slightly higher activity compared to alpha-1 on Na_v_1.5, while six mutants (S12A, T17A, W22A, K25A, W22K, and F24K) were inactive at the tested concentration. The rest of the mutants were less active than alpha-1 on Na_v_1.5. Two mutants (F24A and L11A) were particularly selective toward Na_v_1.5. **Na**_**v**_ **1.6:** Four mutants (T5A, S7A, T10A, and N27A) exhibited slightly higher activity on Na_v_1.6 compared to alpha-1, while two mutants (F8A and N30A) exhibited lower activity. Three mutants (N19A, W22A, and F24A) were inactive at the tested concentration on Na_v_1.6. No mutant was particularly selective toward Na_v_1.6. **Na**_**v**_ **1.7:** The five tested mutants (T5A, S7A, T10A, N27A, and N30A) demonstrated lower activity compared to alpha-1 on Na_v_1.7. No mutant was particularly selective toward Na_v_1.7. **Artemia:** Three mutants (I3A, T5A, and N30A) exhibited similar or slightly higher activity compared to alpha-1 on *Artemia.* Eight mutants (S12A, T17A, K18A, W22A, K25A, F8K, W22K, and F24K) were inactive at the tested concentration, while the rest of the mutants were less active on *Artemia*. In most cases, the results from *Artemia* corroborated electrophysiological measurements from *in vitro* Na_v_ channels. Only T5A and N30A had higher activity, which can be attributed to a higher basal mortality at low concentrations (Figure S2).

**Figure 5:**
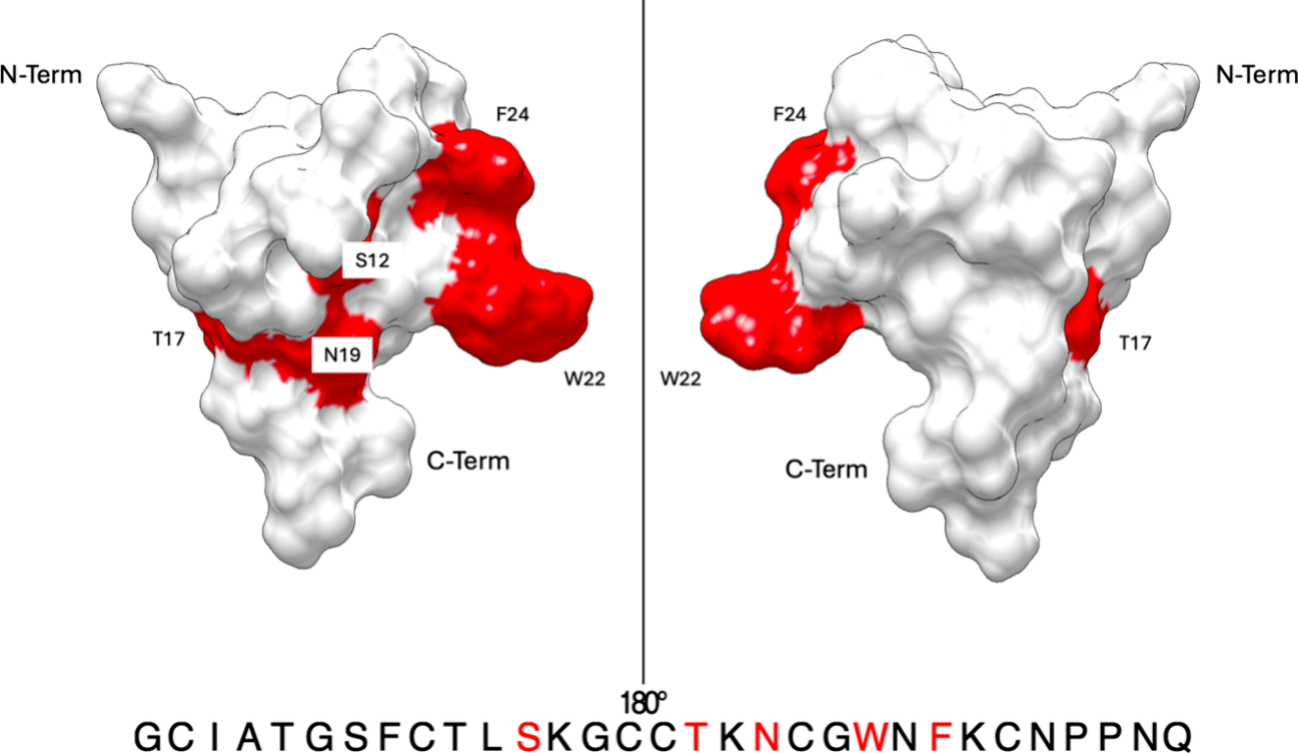
Surface model representation of nemertide alpha-1. The important positions for selectivity on Na_v_ channels are colored in red and labeled. Structures are displayed using UCSF Chimera. PDB ID of nemertide alpha-1: 6ENA.

The modifications in the side chains of the key AA positions resulted in major changes in the physicochemical properties (Table S4). Consequently, the selectivity or affinity for certain ion channels can be affected. These changes could affect the binding to the receptor by altering hydrogen bonds, van der Waals interactions, and electrostatic interactions. In parallel to the alanine scanning, three lysine mutants were also designed based on a 3D structural feature of alpha-1. Phe8, Trp22, and Phe24 make up a hydrophobic patch,^13^ and we hypothesized that this patch could be involved in the binding to the receptor. Therefore, it was decided to alter this patch using a lysine residue, which would decrease hydrophobicity, increase polarity, and increase the number of positive charges. We decided to substitute the native AA for lysine based on its physicochemical properties (Table S4). The results highlighted the importance of W22 and F24 and confirmed results from the alanine scanning previously described. W22K and F24K were inactive on Na_v_1.4, Na_v_1.5, and *Artemia*, while F8K was only inactive on *Artemia.* Hence, W22 and F24 were very important for maintaining the activity of the peptide and would be difficult to replace, whereas F8 appeared more susceptible to modification to steer selectivity and affinity without losing too much activity.

Compared to nemertides alpha-2 to alpha-7, alanine/lysine mutants were mostly deleterious for the activity and only brought improvement in a couple of cases. Additionally, alpha-2 to alpha-7 were the results of single point to multiple point mutations, while alanine/lysine mutants were the result of single-point mutations only. Nemertide alpha-2 to alpha-7 displayed sequence variability on the N-terminus between residues 1 and 10, except for alpha-7, which displayed sequence variability until residue 15. Sequences were highly conserved between residues 10 and 30 on the C-terminal. Alanine/lysine mutant effects appeared in the conserved part of the sequence. Consequently, results underscored the importance of the conserved part of the peptide sequence and underlined the flexibility of the nonconserved part for future sequence modifications.

The current panel focused on four types of Na_v_ channels—BgNa_v_1, Na_v_ 1.3, Na_v_ 1.4, and Na_v_ 1.5—based on the following observations. Αlpha-1 was previously hypothesized to be a potential candidate for the development of novel biopesticides thanks to its remarkable activity on cockroach Na_v_s.^13^ An *in vitro* observation also suggested that nemertide alpha-1’s mode of action on Na_v_ 1.5 could restore the physiological function of sodium channel genetic variants in Brugada syndrome, an inherited cardiac arrhythmia syndrome.^24^ Three analogues of alpha-1 (alpha-4, alpha-5, and alpha-6) exhibited lower EC_50_ compared to alpha-1 on Na_v_ 1.3, Na_v_ 1.4, and Na_v_ 1.5.^14^ These channels are respectively associated with pain/epilepsy, skeletal muscle paralysis, and cardiac arrhythmia, making them an overall interesting panel to investigate possible further drug development.^25,26^

One mutant stood out especially in the present analysis. S12A displayed an interesting activity profile. When screened on a wider panel of ion channels,^14^ S12A proved to be insect-specific (Table 3, Figure S4) despite being 28 times less active than alpha-1. It was inactive on all mammalian ion channels tested and active on most insect ion channels tested (*Blatella germanica*, *Varroa destructor*, *Aedes aegypti*, and *Bombus impatiens*). Surprisingly, S12A was not active on *Drosophila melanogaster* ion channels (Shaker IR). Although the structural explanation behind this effect is currently tentative, the discovery points to S12A as a potential scaffold for biopesticide investigation and development, especially in light of recent encouraging results on alpha-1 biopesticidal effects.^15,16^

**Table 3:** Panel of Mammalian and Insect Ion Channels Tested with S12A at 5 μM^a^

Channel		Channel		Channel	
Kv1.1	–	Cav3.1	–	Nav1.1	–
Kv1.2	–	Cav3.2	–	Nav1.2	–
Kv1.3	–	Cav3.3	–	Nav1.3	–
Kv1.4	–	αβγδ	–	Nav1.4	–
Kv1.5	–	αβδε	–	Nav1.5	–
Kv1.6	–	α3β2	–	Nav1.6	–
Kv2.1	–	α4β2	–	Nav1.7	–
Kv3.1	–	α4β4	–	Nav1.8	–
Kv4.3	–	α7	–	BgNav	**+**
Kv7.1	–	α9α10	–	VdNav	**+**
Kv7.2/Kv7.3	–	ASIC1a	–	AaNav	**+**
Kv10.1	–	ENaC	–	BiNav	**+**
Kv11.1 (hERG)	–	μ-Opoid receptor	–		
Shaker IR	–	TRPV1	–		
KQT-1	–	TRPA1	–		

a “**+**” means tested and produced effect. “–“ means tested but no effect. Both “*” cells contain *Drosophila melanogaster* ion channels.

In conclusion, this work delivers critical insights into the molecular determinants of nemertide alpha-1 activity and selectivity across Na_v_ channels, providing an unprecedented structural map of functional and adaptable regions within this marine peptide scaffold. By pinpointing residues such as S12, T17, N19, W22, and F24, we establish a rational framework for engineering analogues with precision-tuned pharmacological profiles. Most notably, the S12A variant demonstrates exceptional selectivity for invertebrate Na_v_ channels, positioning nemertides as a unique and timely solution for next-generation biopesticides with minimal mammalian off-target risk. In an era of escalating resistance to conventional insecticides and a growing demand for environmentally safe pest control, these findings underscore the urgent need to harness marine natural products for sustainable agriculture. Our study not only advances fundamental toxin biology but also opens a strategic pathway for translating biodiversity into practical, selective bioactive agents.

## Experimental Section

### Synthesis of Alpha-Nemertide Mutants

Peptides were manually assembled on a 0.1 mM scale using Fmoc-based solid-phase peptide synthesis (SPPS) on a custom synthetic Fmoc Gln­(Trt) TentaGel XV HMPA resin. All amino acids were coupled using 6 equiv. To prevent chain aggregation, leucine and serine, respectively, in positions 11 and 12 were coupled as dipeptide Fmoc-Leu-Ser­(ψMe,MePro)-OH. For L11A, S12A, K13A, and T17A, glycine and cysteine, respectively, in positions 14 and 15 were coupled as dipeptide Fmoc-Gly-Cys­(ψ­(Dmp, H)­pro)-OH instead. Peptides were cleaved from the resin using TFA/H_2_O/TIPS (95:2.5:2.5 v/v/v) for 2 h, precipitated using ice-cold ether, redissolved with MeCN/H_2_O (1:1), and lyophilized.

Crude peptides were purified using RP-HPLC on a YMC-Actus Triart Prep C18-S column (250 mm × 20 mm, 10 μm, 120 Å, YMC) and a Kinetex XB-C18 LC column (250 mm × 21.2 mm, 5 μm, 100 Å, Phenomenex) using a gradient from 5% to 95% MeCN in 0.05% TFA.

Peptides were then subjected to oxidative folding based on the protocol developed by Jacobsson et al.,^14^ in GSH/GSSG 2 mM/0.4 mM in 0.1 M NH_4_HCO_3_ (pH 8.5), containing 20% (v/v) DMSO, to subsequently be purified using a YMC-Triart Prep C18-S column (250 × 10 mm, 10 μ, 120 Å, YMC). Fractions (1 min) were collected, and toxin-containing fractions were identified by direct injection into a QToF (Micromass QToF Micro, Waters, MA, USA). Analytical runs were performed using a BioBasic-18 column (150 × 4.6, 5 μ, 300 Å, ThermoFisher) on an HPLC/UV-MS system fit with electrospray ionization (ESI) (Agilent LCMS 1200; Agilent 6130 Quadrupole, St. Clara, USA) and using a gradient from 5% to 95% MeCN in 0.05% FA. An AQUITY Premier CSH C18 column (2.1 × 100 mm, 1.7 μm, 130 Å, Waters Corporation) on an AQUITY Premier UPLC system (Waters Corporation) coupled to a Xevo-G2-XS QToF mass spectrometer (Waters Corporation) was also used for recording monoisotopic masses. A linear gradient from 5% to 95% MeCN 0.1% FA at a flow rate of 0.65 mL/min over 10 min was used for analysis, and the scan window was set to 100–2000 *m*/*z* in positive mode. Pure (>95%, Figure S1), lyophilized, and folded peptide was quantified using IR spectroscopy (DirectDetect, Milipore Corp, MA, USA).

### Microwell *Artemia* Bioassay

Mutant toxicity was assessed with an *Artemia* microwell bioassay,^27^ as a standard estimate for *in vivo* crustacean EC_50_s for these toxins.^13,14^ *Artemia* cysts (Artemio pur, JBL, Neuhofen, Germany) were hatched in a graduated cylinder containing artificial seawater (33 g salt/L, Blue Treasure, SPS Sea Salt, Qingdao, China) prepared in deionized water. Water was aerated at RT for 24 h using a small aquarium air pump. Aliquots of 100 mL test solutions (3 nM to 60 mM nemertide in MQ-water, negative control: 100 mL of MQ-water, positive control: 60 mM alpha-1) were prepared and added in triplicates to flat-bottom 96-well plates (cat. no.: Nunc 260895, Thermo Fisher Scientific, Waltham, MA, USA). A homogeneous number of nauplii (10–15) in 100 μL was harvested and transferred to the 100 μL test solution for a total volume of 200 μL. Incubation was performed for 24 h in the dark. Each well was examined after 24 h with a microscope, and all dead and immobilized nauplii were counted. Finally, all nauplii were sacrificed by incubation with 100 μL of MeOH for 30 min, and the total numbers of nauplii were counted.

### Electrophysiology

For the expression of Na_V_ channels (hNav1.1, rNa_V_1.2, rNa_V_1.3, rNa_V_1.4, hNa_V_1.5, hNa_V_1.6, hNav1.7, and the insect channel BgNa_V_1) in *Xenopus laevis* oocytes, the linearized plasmids were transcribed using the T7 or SP6 mMESSAGE-mMACHINE transcription kit (Ambion, Carlsbad, CA, USA). The harvesting of stage V and VI oocytes from an anaesthetized female *X. laevis* frog was described previously.^28,29^ Oocytes were injected with 50 nL of cRNA at a concentration of 1 ng/nL using a microinjector (Drummond Scientific, Broomall, PA, USA). The oocytes were incubated in a solution containing 96 mM NaCl, 2 mM KCl, 1.8 mM CaCl_2_, 2 mM MgCl_2_, and 5 mM HEPES (pH 7.4), supplemented with 50 mg/L gentamycin sulfate. Two-electrode voltage-clamp recordings were performed at room temperature (18–22 °C) using a Geneclamp 500 amplifier (Molecular Devices, Downingtown, PA, USA) controlled by a pClamp data acquisition system (Axon Instruments, Union City, CA, USA). Whole-cell currents from oocytes were recorded 1–4 days after injection. The bath solution composition was 96 mM NaCl, 2 mM KCl, 1.8 mM CaCl_2_, 2 mM MgCl_2_, and 5 mM HEPES (pH 7.4). Voltage and current electrodes were filled with 3 M KCl. The resistances of both electrodes were kept between 0.8 and 1.5 MΩ. The elicited Na_v_ channel currents were filtered at 1 kHz and sampled at 20 kHz by using a four-pole low-pass Bessel filter. Leak subtraction was performed using a −P/4 protocol. For the electrophysiological analysis of toxins, a series of protocols was applied from a holding potential of −90 mV with a start-to-start interval of 0.2 Hz. Sodium current traces were evoked by 100 ms depolarizations to *V*_max_ (the voltage corresponding to maximum sodium current in control conditions).

To assess the concentration–response relationships, data were fitted with the Hill equation: *y* = 100/[1 + (EC_50_/[toxin])^*h*^], where *y* is the amplitude of the toxin-induced effect (*y* = *I*_30 ms_/*I*_peak_ where *I*_30 ms_ is the current value at 30 ms in the presence of toxin and *I*_peak_ is the peak current amplitude in control conditions), EC_50_ is the toxin concentration at half-maximal efficacy, [toxin] is the toxin concentration, and *h* is the Hill coefficient. Potassium and calcium currents were evoked by a 500 ms depolarization to the voltage corresponding to the maximal activation of the channels in control conditions from a holding potential of −90 mV. Elicited currents were sampled at 1 kHz and filtered at 0.5 kHz. For nAChR experiments, the oocytes were clamped at a holding potential of −70 mV and continuously superfused with ND96 via gravity-fed tubes at 0.1–0.2 mL min^–1^, with 5 min incubation times for the bath-applied peptides. ACh was applied via gravity-fed tubes until a peak current amplitude was obtained (1–3 s), with 1–2 min washout periods between applications. The nAChR were gated by a particular time duration pulse of ACh for the respective nAChR subtype at 2 mL min^–1^. Data were sampled at 500 Hz and filtered at 200 Hz. ASIC1a currents were measured in ND96 solutions at a pH of 7.4 and 4.5, at a holding potential of −70 mV during 400 s, and the data were filtered at 20 Hz. EnaC currents were measured by clamping oocytes at a holding potential of −60 mV and perfusing with low- (1 mM) and high- (96 mM) sodium ND96. Opioid currents were measured by clamping the potential to −70 mV and changing the perfusion solution from ND96 to high-potassium (HK) solution composed of 96 mM KCl, 2 mM NaCl, 1 mM MgCl_2_, 1.8 mM CaCl_2_, and 5 mM HEPES, with a final pH of 7.5. The HK-evoked increase in inward K^+^ currents represents a “basal” K^+^ current (IK, basal). In the presence of HK, 0.2 μM morphine was applied to oocytes and then washed out by HK. TRPV1 and TRPA1 currents were continuously monitored in a perfusing ND96 solution, following a protocol at −90 mV for 400 s. The sampling rate was set at 100 Hz, and the signals were filtered at 20 Hz. One μM capsaicin and 10 μM capsazepine were used for TRPV1. In the TRPA1 experiments, 50 μM mustard oil was used. Peak current amplitude was measured prior to and following the application of the peptide. All data were obtained in at least three independent experiments (*n* ≥ 3). All data were analyzed using pClamp Clampfit 10.0 (Molecular Devices) and Origin 7.5 software (Originlab, Northampton, MA, USA). The use of the frogs was in accordance with license number LA1210239 of the Laboratory of Toxicology & Pharmacology, University of Leuven. All animal care and experimental procedures were in accordance with the guidelines of “European convention for the protection of vertebrate animals used for experimental and other scientific purposes” (Strasbourg, 18.III.1986).

### Data Analysis

Toxicity was calculated as the percentage of dead or immobilized nauplii per well/total number of nauplii per well. Analysis was conducted in R (R Core team: R v.3.2.3 and RStudio 1.1.463) with the package drc^30^ to produce dose–response curves (log–logistic function). Nonlinear regression (curve fit) was used based on a log (concentration) vs response–variable slope (four parameters) model to determine the EC_50_ toxicity of alpha-nemertide mutants. Figures were assembled using the packages pheatmap and ggplot2.^31^

## Supplementary Material



## Notes

Theoretical and experimental molecular mass values for the alpha-nemertide mutants; EC_50_ values of alpha-nemertide mutants in Na_V_1.1–1.7 and BgNav1 and *Artemia salina*; normalized EC_50_ values of alpha-nemertide mutants in Na_V_1.1–1.7 and BgNa_v_1; summary of the changes in physicochemical properties upon modification of the native amino acid with alanine or lysine; purity of alpha-nemertide mutant toxins (Figure S1); dose–response curves of the 20 alpha-nemertides mutants in the *Artemia* microwell assay; heatmap of the normalized EC_50_ from alanine mutants on Na_v_ channels and *Artemia salina*; selectivity screening of mutant S12A (PDF)
