# Supplementary material for: Alanine Mutagenesis Identifies Specific Amino Acids of Nemertide Alpha‑1 Activity and Its Binding to Target Receptors
Source: J Nat Prod. 2025 Dec 17;89(1):95–103. doi: 10.1021/acs.jnatprod.5c01177 (PMC12836348; doi:10.1021/acs.jnatprod.5c01177)
Supplement: Supplementary file 1 [file np5c01177_si_001.pdf]

Supplementary Information: Alanine mutagenesis identifies specific amino acids of nemertide alpha-1 activity and its binding to target receptors

Quentin Laborde[1#], Steve Peigneur[2,3#], Erik Jacobsson[1], Ulf Göransson[1], Jan Tytgat[2*], and Håkan S. Andersson[1,4*]

[1]Pharmacognosy, Department of Pharmaceutical Biosciences, Biomedical Center, Uppsala University, Box 574, SE-751 23, Uppsala, Sweden.

[2]Toxicology & Pharmacology, University of Leuven (KU Leuven), O&N 2, PO Box 922, Herestraat 49, 3000, Leuven, Belgium.

[3]University of Vienna, Faculty of Chemistry, Institute of Biological Chemistry, Währinger Straße 38, 1090, Vienna, Austria

[4]Department of Medical Biochemistry and Biophysics, Karolinska Institutet, 17177 Stockholm, Sweden

# Shared first authorship; *Corresponding authors

Table of Contents

1. Theoretical and experimental molecular mass values for alpha-nemertide mutants (Table S1) 2
2. EC_{50} values of alpha-nemertide mutants in Na_V1.1–1.7 and BgNav1 (*ex vivo*, nM) and Artemia salina (*in vivo*, μM) (Table S2) 3
3. Normalised EC_{50} values of alpha-nemertide mutants in Na_V1.1–1.7 and BgNav1. (Table S3) 4
4. Summary of the changes in physicochemical properties upon modification of the native amino acid with alanine or lysine (Table S4) 5
5. Purity of alpha-nemertide mutant toxins (Figure S1) 6
6. Dose-response curves of the 20 alpha-nemertides mutants in the *Artemia* microwell assay (Figure S2) 8
7. Heatmap of the normalised EC_{50} from alanine mutants on Na_v channels and *Artemia salina*. (Figure S3) 11
8. Selectivity screening of mutant S12A (Figure S4) 12

Tables

Table S1: Theoretical and experimental molecular mass values for alpha-nemertide mutants. Theoretical and experimentally determined deconvoluted (3z) masses. The 3z ion was chosen since it falls within the calibrated mass range for the QToF used.

Peptide	Expected (Da)	Experimental (Da)	Delta (Da)	Delta (ppm)
**I3A**	3265.2970	3265.33	0.0341	10.4
**T5A**	3277.3330	3277.35	0.0200	6.1
**S7A**	3291.3490	3291.39	0.0392	11.9
**F8A**	3231.3120	3231.3561	0.0441	13.6
**T10A**	3277.3330	3277.3683	0.0353	10.8
**L11A**	3265.2970	3265.3311	0.0341	10.4
**S12A**	3291.3490	3291.3816	0.0326	9.9
**K13A**	3250.2860	3250.3326	0.0466	14.3
**T17A**	3277.3330	3277.359	0.0260	7.9
**K18A**	3250.2860	3250.3326	0.0466	14.3
**N19A**	3264.3380	3264.3687	0.0307	9.4
**W22A**	3192.3010	3192.3351	0.0341	10.7
**N23A**	3264.3380	3264.3672	0.0292	8.9
**F24A**	3231.3120	3231.3357	0.0237	7.3
**K25A**	3250.2860	3250.3194	0.0334	10.3
**N27A**	3264.3380	3264.3585	0.0205	6.3
**N30A**	3264.3380	3264.3585	0.0205	6.3
**F8K**	3288.3700	3288.4119	0.0419	12.7
**W22K**	3249.3590	3249.3828	0.0238	7.3
**F24K**	3288.3700	3288.4119	0.0419	12.7

Table S2: EC_{50} values of alpha-nemertide mutants in $Na_V1.1–1.7$ and BgNav1 (*ex vivo*, nM) and Artemia salina (*in vivo*, µM).

Peptide	BgNav1	Nav1.1	Nav1.2	Nav1.3	Nav1.4	Nav1.5	Nav1.6	Nav1.7	Artemia
Alpha-1	8.6 ± 2.9	124.1 ± 28.7	359.6 ± 89.8	135.4 ± 76.3	145.5 ± 57.5	138.3 ± 25.5	240.4 ± 22.3	75.6 ± 33.9	1.2 ± 0.15
I3A	49.7 ± 9.1	-	-	186.3 ± 32.4	375.8 ± 82.8	701.4 ± 115.9	-	-	1.2 ± 0.47
T5A	45.6 ± 3.7	124.1 ± 29.6	379.7 ± 65.3	127.2 ± 43.7	112.9 ± 16.9	142.5 ± 43.3	107.6 ± 39.5	114.1 ± 18.5	0.53 ± 0.98
S7A	35.9 ± 13.3	115.2 ± 39.7	885.1 ± 68.1	93.4 ± 14.7	90.6 ± 37.0	146.6 ± 31.5	130.6 ± 46.7	136.3 ± 6.6	2.2 ± 3.2
F8A	68.7 ± 12.8	58.9 ± 23.5	560.1 ± 73.2	458.6 ± 61.8	818.1 ± 31.7	250.3 ± 32.3	446.7 ± 92.4	-	7.7 ± 1.1
T10A	155.4 ± 131.7	96.3 ± 21.7	267.6 ± 41.2	418.9 ± 92.4	117.1 ± 51.1	110.2 ± 9.4	112.6 ± 27.9	190.7 ± 6.2	5.2 ± 1.5
L11A	298.2 ± 35.2	-	-	201.5 ± 47.9	704.2 ± 57.4	140.5 ± 18.0	-	-	19.0 ± 0.72
S12A	281 ± 75.0	-	-	n.a	n.a	n.a	-	-	n.a
K13A	386.3 ± 32.4	-	-	379.4 ± 91.0	5514.1 ± 154.5	595.1 ± 20.4	-	-	25.0 ± 1.5
T17A	825.9 ± 4.0	-	-	n.a	n.a	n.a	-	-	n.a
K18A	32.9 ± 10.9	-	-	675.4 ± 15.1	667.1 ± 17.9	373.1 ± 152.7	-	-	n.a
N19A	19.2 ± 7.3	n.a	n.a	n.a	n.a	534.2 ± 60.9	n.a	-	9.2 ± 18
W22A	2004 ± 188.0	4721.2 ± 595.5	n.a	n.a	n.a	n.a	n.a	-	n.a
N23A	19.5 ± 3.4	406.5 ± 144.1	1674.7 ± 187.4	183.9 ± 37.6	229.6 ± 14.6	307.1 ± 12.5	-	-	1.5 ± 2.9
F24A	1046.5 ± 196.1	n.a	n.a	779.2 ± 77.3	n.a	577.1 ± 28.8	n.a	-	21.0 ± 3.4
K25A	454.7 ± 69.4	-	-	445.7 ± 52.4	919.8 ± 79.7	n.a	-	-	n.a
N27A	48.3 ± 2.8	98.9 ± 9.5	354.4 ± 58.6	465.7 ± 21.3	58.3 ± 28.2	320.0 ± 163.8	185.0 ± 24.4	972.6 ± 112.4	5.6 ± 4.8
N30A	137.9 ± 6.2	111.3 ± 32.5	n.a	2759.5 ± 458.4	99.0 ± 26.7	167.3 ± 66.5	765.0 ± 61.3	1023.4 ± 2.8	0.52 ± 0.25
F8K	91.9 ± 8.4	-	-	467.2 ± 64.9	1203.5 ± 147.2	223.9 ± 6.33	-	-	n.a
W22K	1051.0 ± 178.7	-	-	n.a	n.a	n.a	-	-	n.a
F24K	599.6 ± 86.7	-	-	n.a	n.a	n.a	-	-	n.a

Table S3: Normalised EC_{50} values of alpha-nemertide mutants in $Na_V1.1$–1.7 and BgNav1. EC_{50} values are normalised based on the lowest value for each mutant (See Table S1). n.a: not active at tested concentration.

Peptide	BgNav1	Nav1.1	Nav1.2	Nav1.3	Nav1.4	Nav1.5	Nav1.6	Nav1.7
Alpha-1	**1.0**	14.4	41.8	15.7	16.9	16.1	28.0	8.8
I3A	**1.0**	-	-	3.7	7.6	14.1	-	-
T5A	**1.0**	2.7	8.3	2.8	2.5	3.1	2.4	2.5
S7A	**1.0**	3.2	24.7	2.6	2.5	4.1	3.6	3.8
F8A	1.2	**1.0**	9.5	7.8	13.9	4.2	7.6	-
T10A	1.6	**1.0**	2.8	4.3	1.2	1.1	1.2	2.0
L11A	2.1	-	-	1.4	5.0	**1.0**	-	-
S12A	**1.0**	-	-	n.a	n.a	n.a	-	-
K13A	**1.0**	-	-	1.0	14.5	1.6	-	-
T17A	**1.0**	-	-	n.a	n.a	n.a	-	-
K18A	**1.0**	-	-	20.5	20.3	11.3	-	-
N19A	**1.0**	n.a	n.a	n.a	n.a	27.8	n.a	-
W22A	**1.0**	n.a	n.a	n.a	n.a	n.a	n.a	-
N23A	**1.0**	20.8	85.9	9.4	11.8	15.7	-	-
F24A	1.8	n.a	n.a	1.4	n.a	**1.0**	n.a	-
K25A	**1.0**	-	-	1.0	2.1	n.a	-	-
N27A	**1.0**	2.0	7.3	9.6	1.2	6.6	3.8	20.1
N30A	1.4	1.1	n.a	27.9	**1.0**	1.7	7.7	10.3
F8K	**1.0**	-	-	5.1	13.1	1.0	-	-
W22K	**1.0**	-	-	n.a	n.a	n.a	-	-
F24K	**1.0**	-	-	n.a	n.a	n.a	-	-

Table S4: Summary of the changes in physicochemical properties upon modification of the native amino acid with alanine or lysine. "/": No change. Ser: serine, Ala: alanine, Thr: threonine, Trp: tryptophan, Phe: phenylalanine, Lys: lysine, Asn: asparagine, Gln: glutamine. *: property of the substituted AA.

Modification	Polarity	Hydrophobicity	Side chain volume	Chemical group	Number of charges	Hydrogen donor or acceptor*
Ser → Ala	Decrease	Increase	/	Hydroxyl to aliphatic	/	Donor
Thr → Ala	Decrease	Increase	Slightly reduced	Hydroxyl to aliphatic	/	Donor
Trp → Ala	/	/	Highly reduced	Aromatic to aliphatic	/	Donor
Phe → Ala	/	/	Highly reduced	Aromatic to aliphatic	/	Neither donor or acceptor
Lys → Ala	Decrease	Increase	Highly reduced	Basic to aliphatic	Decrease number of positive charges	Donor
Asn → Ala	Decrease	Increase	Slightly reduced	Amide to aliphatic	/	Donor
Gln → Ala	Decrease	Increase	Moderately reduced	Amide to aliphatic	/	Donor
Phe → Lys	Increase	Decrease	Slightly reduced	Aromatic to basic	Increase number of positive charges	Donor
Trp → Lys	Increase	Decrease	Slightly reduced	Aromatic to basic	Increase number of positive charges	Donor

Figures

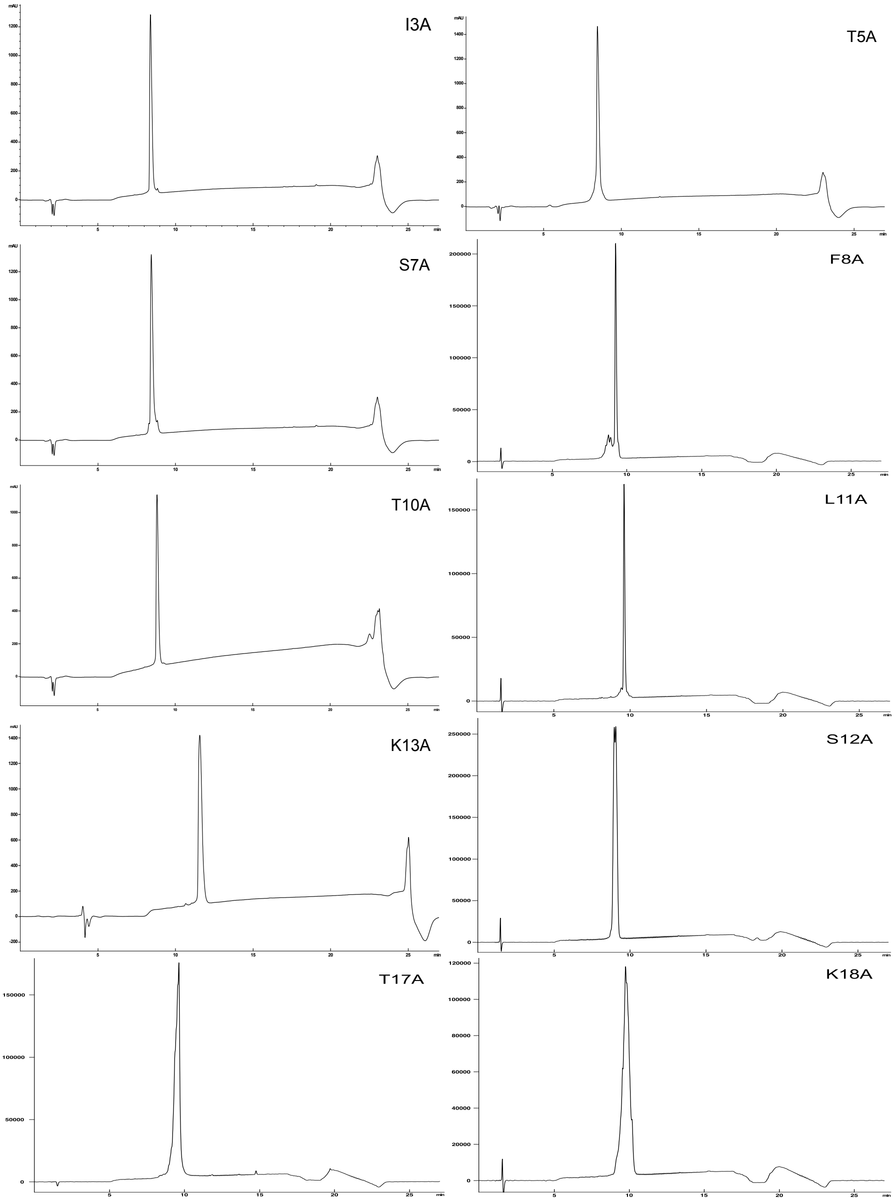

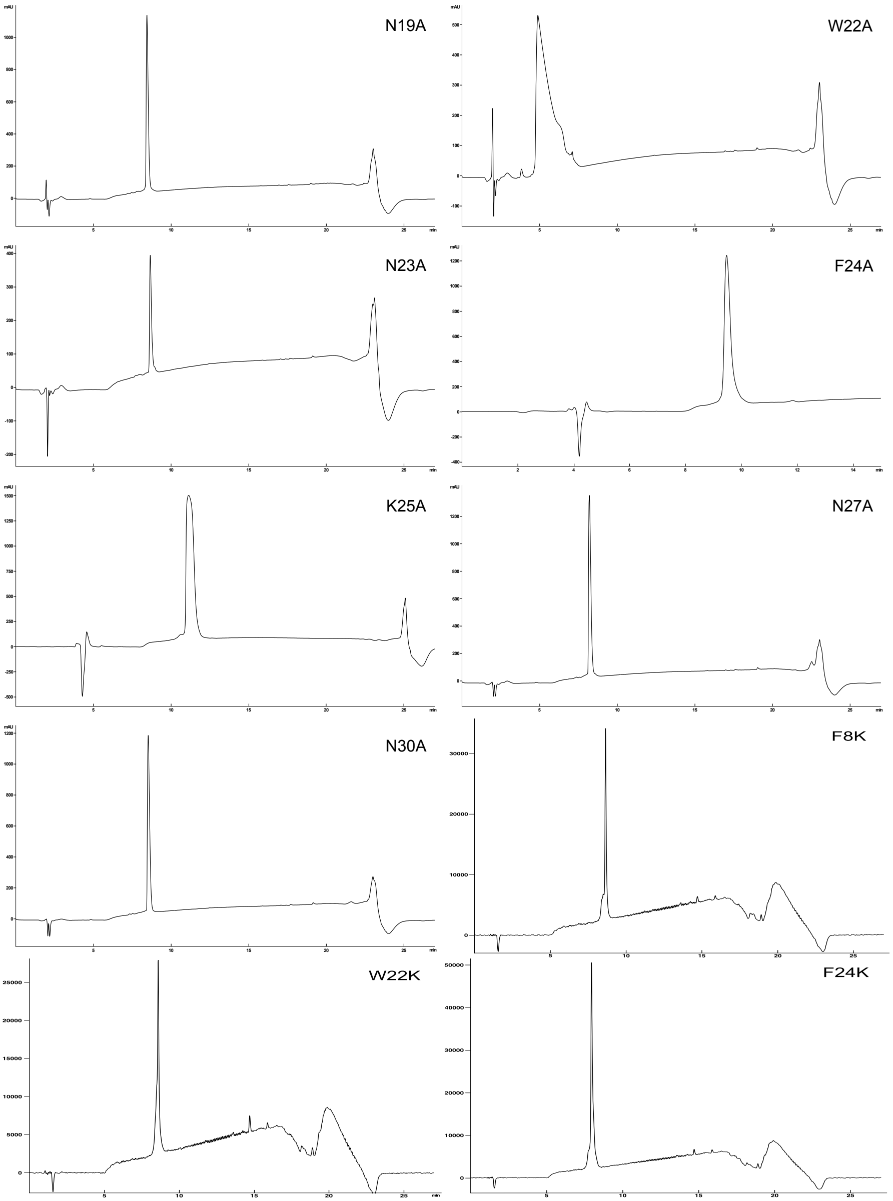

Figure S1: Purity of alpha-nemertide mutant toxins. Absorbance at 215 nm. Note that several systems were used for the purity determination, and that the relative intensity cannot be compared directly between samples. Agilent LCMS 1200: I3A, T5A, S7A, T10A, K13A, N19A, W22A, N23A, F24A, K24A, N27A, N30A. Shimadzu LC-20: F8A, L11A, S12A, T17A, K18A, Q31A, F8K, W22K, F24K.

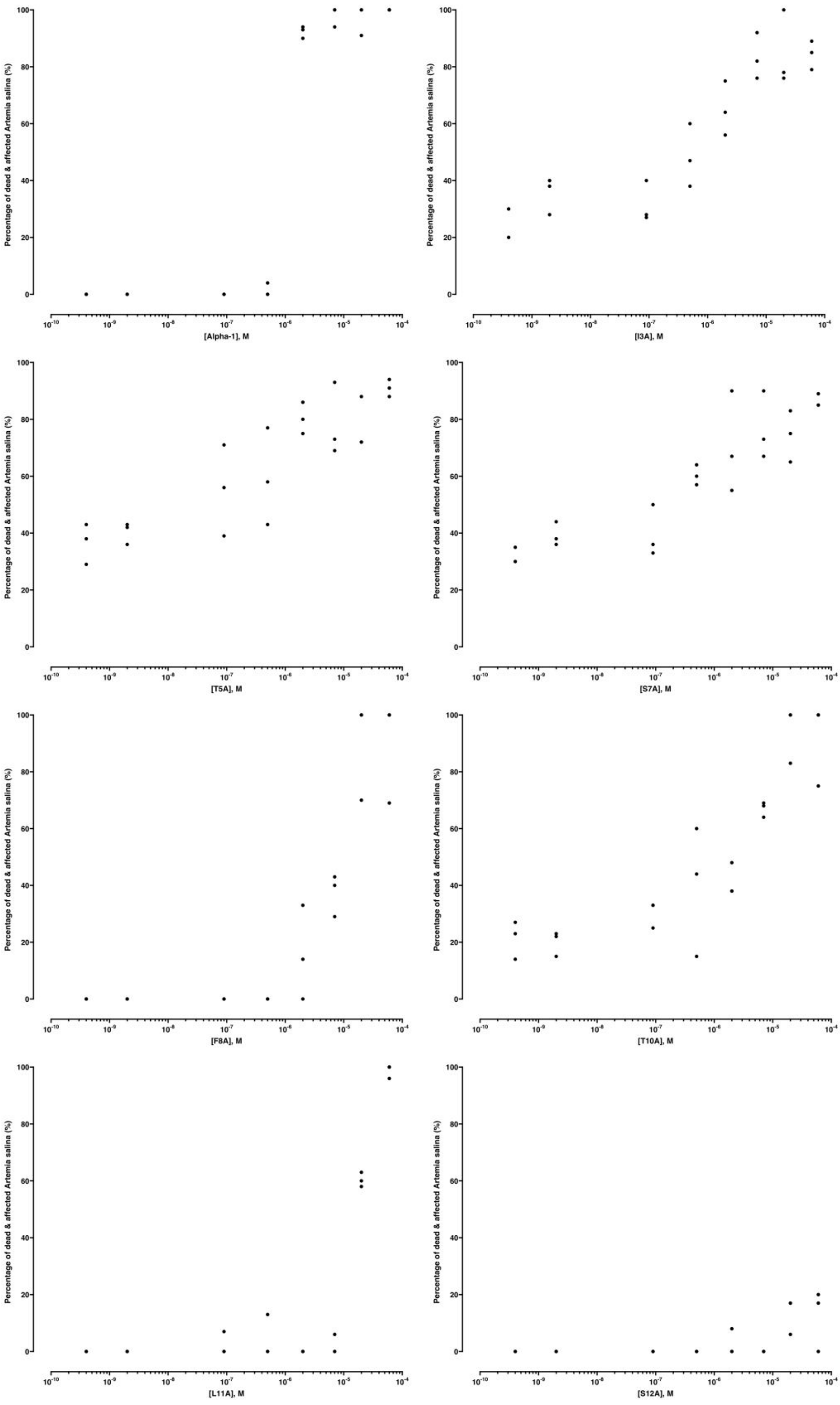
Percentage of dead & affected Artemia salina (%)
[Alpha-1], M
[I3A], M
[T5A], M
[S7A], M
[F8A], M
[T10A], M
[L11A], M
[S12A], M

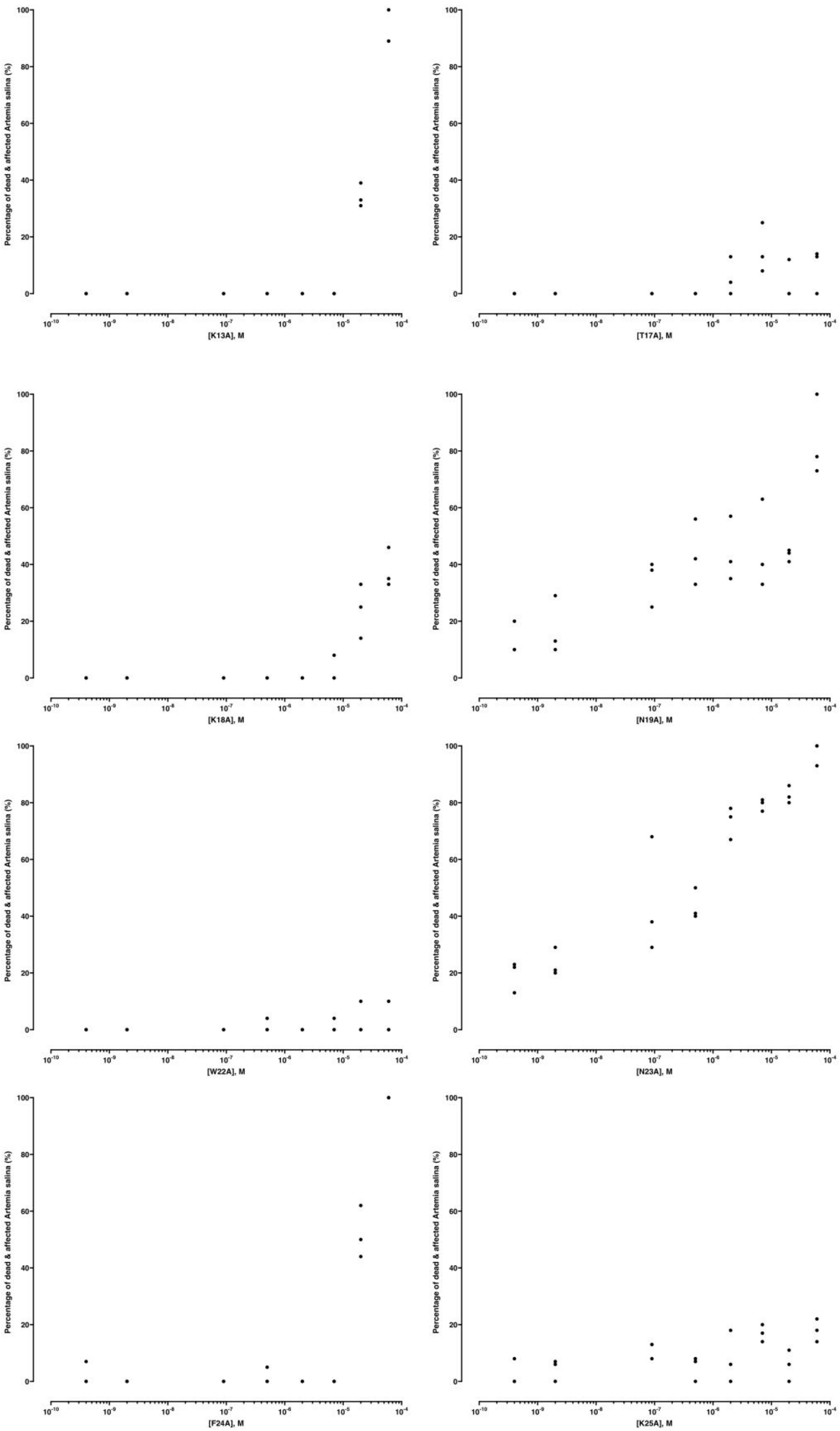
Percentage of dead & affected Artemia salina (%)
[K13A], M
[T17A], M
[K18A], M
[N19A], M
[W22A], M
[N23A], M
[F24A], M
[K25A], M

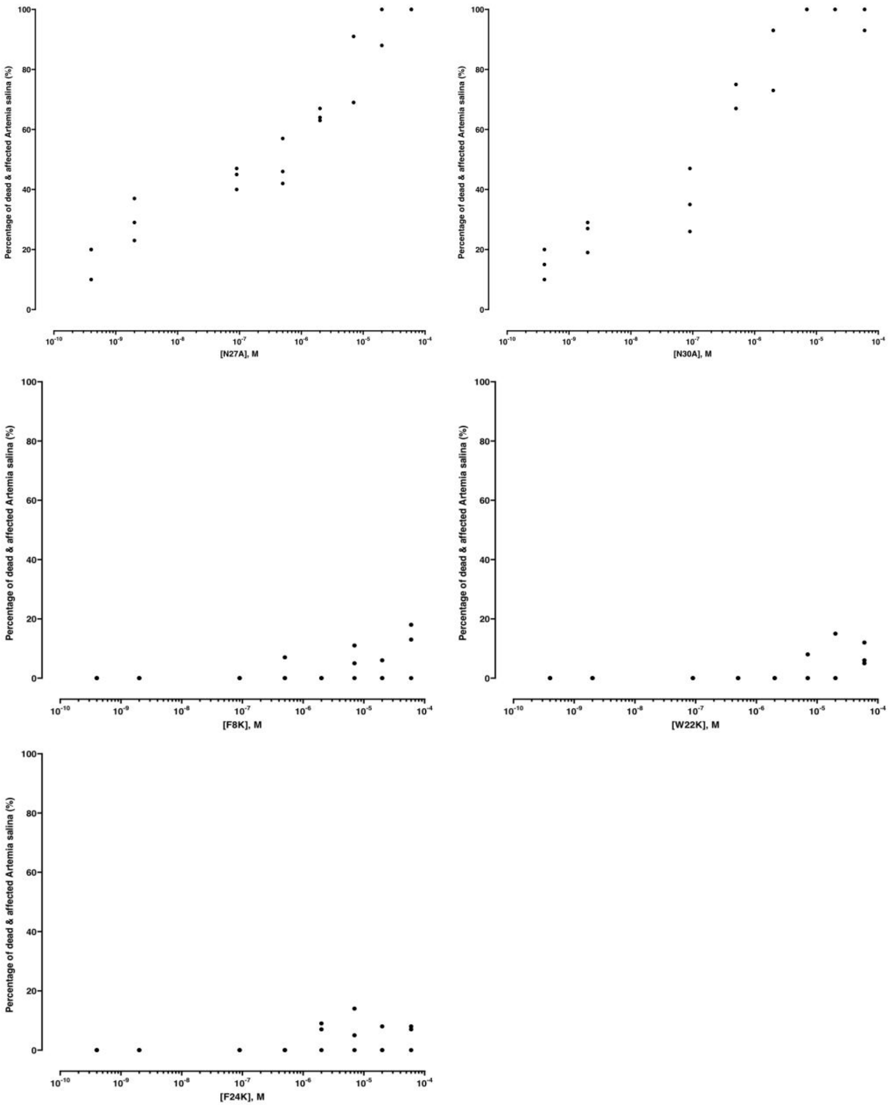

Figure S2: Dose-response curves of the 20 alpha-nemertides mutants in the *Artemia* microwell assay. All experiments were performed in triplicate. All data points are shown in the graphs as dots.

	BgNav1	Nav1.1	Nav1.2	Nav1.3	Nav1.4	Nav1.5	Nav1.6	Nav1.7	Artemia
	10.3 nM	124.1 nM	359.6 nM	135.4 nM	184.0 nM	138.3 nM	240.4 nM	76.5 nM	1.2 µM
a-1	1.0	1.0	1.0	1.0	1.0	1.0	1.0	1.0	1.0
I3A	4.8	-	-	1.4	2.0	5.1	-	-	1.0
T5A	4.4	1.0	1.1	1.1	1.6	1.0	2.2	1.5	2.3
S7A	3.5	1.1	2.5	1.4	2.0	1.1	1.9	1.8	1.8
F8A	6.7	2.2	1.6	3.4	4.4	1.8	1.9	-	6.4
T10A	15.1	1.3	1.3	3.1	1.6	1.2	2.1	2.5	4.3
L11A	29.0	-	-	1.5	3.8	1.0	-	-	15.8
S12A	27.3	-	-	NA	NA	NA	-	-	NA
K13A	37.5	-	-	2.8	30.0	4.3	-	-	20.8
T17A	80.2	-	-	NA	NA	NA	-	-	NA
K18A	3.2	-	-	5.0	3.6	2.7	-	-	NA
N19A	1.9	NA	NA	NA	NA	3.9	NA	-	7.7
W22A	194.6	NA	NA	NA	NA	NA	NA	-	NA
N23A	1.9	3.3	4.7	1.4	1.2	2.2	-	-	1.2
F24A	101.6	NA	NA	5.8	NA	4.2	NA	-	17.5
K25A	44.1	-	-	3.3	5.0	NA	-	-	NA
N27A	4.7	1.3	1.0	3.4	3.2	2.3	1.3	12.7	4.7
N30A	13.4	1.1	NA	20.4	1.9	1.2	3.2	13.4	2.3
F8K	8.9	-	-	3.5	6.5	1.6	-	-	NA
W22K	102.0	-	-	NA	NA	NA	-	-	NA
F24K	58.2	-	-	NA	NA	NA	-	-	NA

Colour scale: 200, 100, 1, 20, 40

Figure S3: Heatmap of the normalised EC_{50} from alanine mutants on Na_v channels and *Artemia salina*. White cells represent mutants exhibiting activity comparable to that of alpha-1. Blue cells (light blue to dark blue) represent mutants with a lower activity than alpha-1. Red cells (light red to dark red) represent mutants with a higher activity than alpha-1. Black cells represent mutants with no activity compared to alpha-1. White cells with a dash "-" represent missing values. Values indicate how many folds the mutant differs from a-1. Each column has been normalised to alpha-1 EC_{50} for each channel. Scales are different between the blue (1 to 200) and the red part (1 to 40). N.A: Not active at the highest tested concentration (50 µM for electrophysiology measurements and 60 µM for *Artemia salina* measurements).

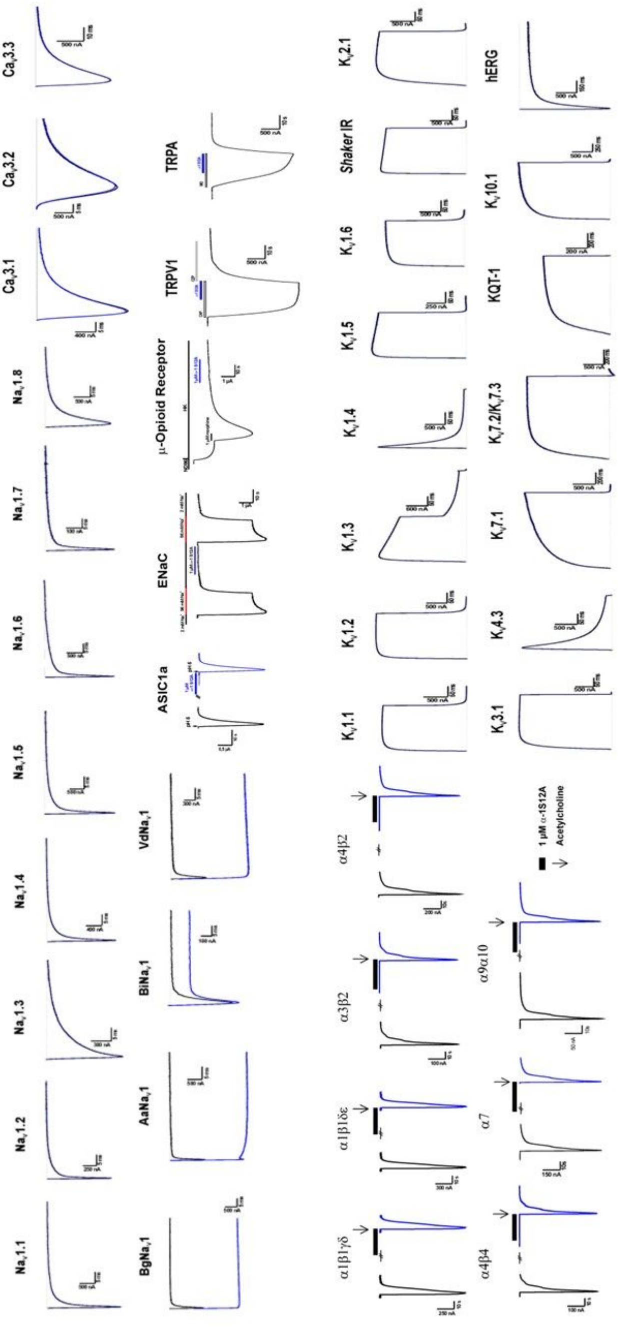

Figure S4: Selectivity screening of mutant S12A shows that this peptide is insect-selective since, at a concentration of 5 μM only insect Na$_V$ channels are modulated.
